# Interrogating the Molecular Mechanisms of Hyperuricemic Nephropathy

**DOI:** 10.34067/KID.0000001181

**Published:** 2026-04-30

**Authors:** Timothy D. Cummins

**Affiliations:** Division of Nephrology and Hypertension, Department of Medicine, University of Louisville, Louisville, Kentucky

**Keywords:** cytokines, fibrosis, hypoxia, interstitial fibrosis, ischemia, ischemia-reperfusion, nephropathy, renal fibrosis, tubule cells

Hyperuricemia arises because of an imbalance between uric acid production and excretion affected by various factors, such as genetics, diet, fructose and purine metabolism, and innate signaling mechanisms. Approximately two thirds of uric acid excretion occurs through the kidney in tightly regulated processes involving glomerular filtration, tubular reabsorption, secretion, and postsecretory reabsorption mediated by an array of transporters.^1^ Dysfunction of these processes contributes to sustained hyperuricemia. Hyperuricemia negatively affects the kidney through both crystal-dependent and crystal-independent mechanisms, contributing to progressive renal injury and dysfunction.^2^ Previous work by others suggests involvement of distinct miRNA modulation of hypoxia inducible factor-1*α* (HIF-1*α*) in tubule cell injury and diabetic nephropathy,^3^ yet detailed molecular mechanisms of hyperuricemic nephropathy (HN) remain to be elucidated. In this issue of *Kidney360*, Li *et al*. provide new molecular insights into HN by elucidating a HIF-1*α*/miR-295/factor inhibiting HIF-1*α* (FIH-1) feedback loop that impinges on tubular fibrosis.^4^

Using a murine model of HN, the authors demonstrate elevated abundance of miR-295 alongside other miRNAs, accompanied by robust activation of HIF-1*α*. In conjunction, they demonstrate direct binding of HIF-1*α* to the miR-295 promoter in renal tubule cell models after uric acid induction, conferring HN-like features. These data illustrate the proposition that HIF-1*α* inhibition can be repressed by elevation of miR-295 and that HIF-1*α* can directly affect its own activation in a molecularly balanced feedback loop that tunes each signal from miR-295 through HIF-1*α* to elicit cellular effects. Inhibition of miR-295 induced features of tubular fibrosis in HN mice, suggesting a regulatory function in tubulointerstitial fibrosis and HN. Conversely, supplementation of miR-295 mitigated tubulointerstitial fibrosis in HN. Collectively, the data suggest that miR-295 exerts a protective effect against tubule fibrosis in mouse models of HN. The authors follow this up with a series of *in vivo* experiments showing miR-295 interacts with the 3ʹ-untranslated region of FIH-1 mRNA with the potential to repress inhibitory effects of FIH-1 on HIF-1*α*. The authors then perform *in vitro* experiments in human tubule cell cultures illustrating that inhibition of FIH-1 decreases cell death markers and diminishes uric acid induced expression of inflammatory fibrotic cytokine RNA for TGF-*β*, connective tissue growth factor, and FGF-1.

Taken together, the data presented in this work suggest a positive feedback tuning loop where miR-295 represses FIH-1 with HIF-1*α* situated between these two molecules and having the capacity to bind the promoter region of miR-295 to modulate the signals from miR-295 and FIH-1 indirectly. Through a series of molecular interactions between microRNA (miR-295), mRNA (HIF-1*α* and FIH-1), and proteins (HIF-1*α* and FIH-1), a delicate and intricate network of post-transcriptionally regulated functions affects biological outcomes in the fibrotic mouse model of HN. Of note, other regulatory mechanisms could be at play to integrate and finely tune alternate signals outside ischemia and hypoxia stress signals potentially driving the miR-295/HIF-1*α*/FIH-1 feedback loop because other miRNA and input signals were not thoroughly investigated in this specific investigation but were identified in the initial screen. In addition, many facets of this feedback loop will need deeper probing in different models of nephropathy and particularly, in human samples where possible and appropriate. The implications of this feedback loop (miR-295/HIF-1*α*/FIH-1) provide the impetus to conduct more in-depth and targeted studies in related but different nephropathy contexts as well. Overall, the findings in this work suggests targeted investigation and detailed interrogation of the miR-295/HIF-1*α*/FIH-1 feedback loop in humans could fill some of the gaps in our understanding of the molecular mechanisms of HN in the clinic. In particular, mechanistic components of this pathway could be exploited as targets for the development of targeted therapies.
